# Acculturation is associated with older Turkish immigrants’ self-management abilities

**DOI:** 10.1186/s12889-019-7471-0

**Published:** 2019-09-05

**Authors:** Jane M. Cramm, Anna P. Nieboer

**Affiliations:** Department of Social Medical Sciences, Erasmus School of Health Policy and Management, Erasmus University Rotterdam, P.O. Box 1738, 3000 Rotterdam, DR The Netherlands

**Keywords:** Acculturation, Immigrants, Self-management, Ageing, Integration

## Abstract

**Background:**

The few previous studies investigating acculturation and self-management have suggested that increased participation in (or adaptation to) the host culture is associated with better health and disease management. However, research on the relationship between acculturation strategies (attachment to the Dutch and Turkish cultures) and broader self-management abilities among older Turkish immigrants in the Netherlands is lacking. This study aimed to investigate this relationship in this population.

**Methods:**

Turkish immigrants aged > 65 years and residing in Rotterdam, the Netherlands (*n* = 2350), were identified using the municipal register. In total, 680 respondents completed the questionnaire (32% response rate).

**Results:**

The average age of the respondents was 72.90 (standard deviation, 5.02; range, 66–95) years and 47.6% of respondents were women. The majority (80.3%) of respondents reported having low educational levels. Women, single individuals, less-educated respondents, and those with multimorbidity experienced lower levels of attachment to the Dutch culture and reported poorer self-management abilities. Slightly stronger relationships were found between self-management and attachment to the Dutch culture than attachment to the Turkish culture. Multimorbidity negatively affected the self-management abilities of older Turkish people living in the Netherlands.

**Conclusions:**

The study findings indicate that especially attachment to the Dutch culture matters for the self-management abilities of older Turkish immigrants in the Netherlands. Given the high prevalence of multimorbidity in this population, investment in their self-management abilities is expected to be beneficial. Special attention is needed for women, single individuals, less-educated people, and those with multimorbidity. Interventions aiming to better integrate these groups into Dutch society are also expected to be beneficial for their self-management abilities.

## Background

Health care systems worldwide are overwhelmed in dealing with the consequences of ageing populations, and the effects of preventive care remain limited [1, 2]. In the Netherlands, the proportion of older Turkish immigrants is increasing much more rapidly than that of older Dutch natives [3]. Furthermore, the prevalences of chronic diseases [4], poor health [5], depression [6], and health care utilisation [7] are greater among Turkish people than among natives in the Netherlands, making older Turkish immigrants a particularly vulnerable group. The same picture emerges in the rest of Europe; chronic diseases, depressive symptoms, functional limitations, and health care use are much more prevalent among immigrant populations than among natives [8, 9]. The rapid increase in the prevalence of chronic diseases among older (immigrant) populations is an important factor underlying the increased demand for health care services and constraints on the organisation and delivery of care in Europe [9, 10]. Increasing self-management abilities to prevent or reduce early functional loss could partially relieve this burden on the health care system by allowing Turkish immigrants to better maintain independent and autonomous lifestyles for longer periods of time [11].

Investing in older people’s self-management abilities is expected to help prevent further declines in functioning and well-being [12, 13]. Most such efforts target the physical health aspects of ageing and dealing with chronic diseases (e.g. treatment adherence, physical exercise, and healthy diet); the latest evidence, however, shows that care and support should also target broader self-management abilities to maintain overall well-being, such as initiative taking, investment behaviour (e.g. maintaining contact with loved ones, pursuing interests, and keeping busy), and self-efficacy (e.g. belief in one’s ability to achieve goals and express care for others) [14, 15]. Our recent study showed the importance of these broader self-management abilities in addition to health behaviours for physical health, depressive symptoms, and well-being among Turkish older immigrants in the Netherlands, especially among chronically ill individuals [16]. Given the vulnerability of older Turkish people in the Netherlands, enhancement of this group’s broader self-management abilities is expected to help them deal with the ageing process. Such efforts require an increased understanding of factors that influence self-management abilities; who are good self-managers and who are not? How can we improve individuals’ self-management abilities? The degree to which older Turkish people adopt new practices, such as self-management abilities, in their personal behavioural repertoires may vary substantially depending on the level of integration in the new cultural environment. Currently, however, we lack research on this issue.

One aspect that deserves closer examination is acculturation [17–21]. Acculturation has been associated with significant changes in self-management (e.g. achievement of self-efficacy), healthier behaviours, and overall health among immigrants [17, 22–25]. Rather than being conceptualised as a process by which people lose connections to their original culture [26], acculturation should be conceived in terms of individuals’ negotiation of two cultural entities [27, 28]. Responding to distinct sets of norms from the original and host cultures, acculturating individuals develop their own interpretations of appropriate values, customs, and practices as they negotiate between cultural contexts [29]. People have greatly variable abilities to function in new cultural environments [30], and they may seek different levels of attachment to and involvement in a host culture and their culture(s) of origin [31]. Therefore, acculturation is usually defined along two dimensions, expressing the degree of contact and participation in the larger ‘new’ society and the maintenance of the ‘old’ heritage culture and identity [32–34]. Individuals may adapt to a new environment without giving up their adherence to the culture of origin [35–39]. The assumption that acculturation is a bidimensional process has been tested in a few studies, and the results have supported the bidimensional model [40, 41]. Four acculturation strategies have been identified [34, 42].

### Four acculturation strategies

If the situation occurs that older immigrants do not wish to maintain their cultural identity and rather seek daily interaction with the host culture, they are following the *assimilation* strategy [43]. If the opposite occurs and older immigrants rather maintain their cultural identity and avoid any interaction with the host culture, they are following the *separation* strategy. Older immigrants who are interested in both cultures use the *integration* strategy in which they maintain their original culture while at the same time engage in daily interactions with the host culture. Integration may take various forms and degrees of integration, including alternation between and merging of cultural ways [42]. Finally, the *marginalisation* strategy refers to situations in which older immigrants have lost the ability and interest to connect with their original culture as well as the host culture. The assessment of these acculturation strategies is a core feature of acculturation research (e.g. [39, 43, 44–46]), and variations in these strategies have been related to variations in acculturation experience and to adaptation (e.g. [47]).

One could reason that those following the integration strategy of acculturation are better self-managers because they retain valued heritage-culture features while selectively and skilfully adopting new cultural behaviours and beliefs facilitating adjustment. In other words, the skills required to adjust to and integrate into the host culture while holding on to the heritage culture are also beneficial in terms of the ability to take initiative and to invest using a long-term perspective (broader self-management abilities). Similarly, biculturalism or bicultural competence involves the appropriate application of dual modes of social behaviour (e.g. communication skills) to enhance self-determination and adjustment in different cultural contexts [47, 48]. Furthermore, integration, biculturalism, and bicultural competence are associated with the alternation model of second culture acquisition, in which positive bicultural adjustment results from the alteration of behaviours for different contexts, formation of meaningful bicultural relationships, and valuing and understanding of both cultures [30, 49, 50], which facilitate the expression of care for others and investment in social relationships. Those who successfully apply dual modes of social behaviour and develop attachment to the host culture, as well as the culture of origin, are therefore also expected to be better self-managers [51, 52]. Those who follow the assimilation strategy also successfully adopt new cultural behaviours and beliefs, which is expected to be beneficial to their self-management abilities; those who more successfully adjust to a different cultural context are expected to have a stronger belief in their ability to achieve certain goals in life and to adopt a more positive attitude. Hence, they are more likely to report stronger self-efficacy skills and hold a more positive frame of mind.

The few studies investigating acculturation and self-management [51, 52] have suggested that increased participation in (or adaptation to) the host culture (i.e. integration and assimilation) is associated with better health and disease management compared with adoption of the marginalisation and separation acculturation strategies. However, research on the relationship between acculturation strategies (attachment to the Dutch as well as the Turkish culture) and broader self-management abilities among older Turkish immigrants residing in the Netherlands is lacking. This study thus aimed to investigate this relationship in this population, and to examine differences in acculturation strategies and self-management between sub-groups of older Turkish immigrants (men and women, those with high and low education levels, those living alone and those who are married, and those with and without multimorbidity).

## Methods

### Data collection

For this cross-sectional study community-dwelling older Turkish people (aged ≥65 years) residing in Rotterdam, the Netherlands, were identified using the municipal register (*n* = 2350). Respondents were asked to fill in a questionnaire provided in Dutch and Turkish in 2015 and the first 2 months of 2016. Questionnaire were first sent via post and non-respondents received a postal reminder. After these attempts via mail we used at least two house visits to reach our respondents (interviewers both Dutch and Turkish). A total of 213 were ineligible due to change of address (*n* = 110), serious medical issue or death (*n* = 102), and non-Turkish ethnic background (*n* = 1). Of the 2137 eligible respondents a total of 680 filled in the questionnaire leading to a 32% response rate.

### Ethical approval

According to the rules laid down in the Dutch law and followed by the Central Committee on Research Involving Human Subjects (CCMO), the current study did not fall within the scope of the Medical Research Involving Human Subjects Act and therefore did not require prior review by an accredited medical research and ethics committee or the CCMO. All participating respondents were informed about the study and they were assured that participation was completely anonymous and voluntary prior to providing consent.

### Measures

Professional native Turkish translators living in the Netherlands translated the questionnaire (which contained items from multiple instruments and those eliciting background characteristics) into Turkish. All of the translators were official and certified, and studied Turkology, ensuring high-quality translation. The procedure was as follows: a first translator translated the Dutch instrument into Turkish. A second translator then checked all aspects of the initial translation (e.g. spelling, grammar, terminology, and cultural interpretation of words). A third translator then performed back translation to ensure that the items had been translated properly. Finally, the questionnaire was tested among older native Turkish people to ensure content validity for the target population. The Turkish versions of the included measures are presented in the appendix.

The Psychological Acculturation Scale [53, 54] was used to assess attachment to the Dutch and Turkish cultures. This scale was originally developed to assess individuals’ sense of emotional attachment to, belonging within, and understanding of the Anglo American and Latino-Hispanic cultures [53], and was later used to measure psychological acculturation among Moroccan people in the Netherlands. The Dutch–Moroccan psychological acculturation subscale showed a good fit to the data in both populations. Overall, this adapted instrument demonstrated strong psychometric properties in previous studies [53, 54]. In the current study, the Dutch and Turkish Psychological Acculturation Subscales (D-PAS and T-PAS, respectively) showed good reliability (Cronbach’s alpha, 0.83 and 0.86, respectively). In addition to using the D-PAS and T-PAS to investigate attachment to the home and host cultures, in line with previous research [55–57], we constructed the four acculturation strategies by subjecting the home and host culture scales to a median split [36, 58–60]. After calculating the median score on the home (T-PAS) and host culture (D-PAS) of the entire population we now know the acculturation strategy of each individual;

- T-PAS and D-PAS of an individual both above the median of the total population represents the integration strategy
- T-PAS below the median and D-PAS above the median represents the assimilation strategy
- T-PAS above the median and D-PAS below the median represents separation the strategy
- T-PAS and D-PAS both above the median represents the marginalization strategy

Self-management abilities were assessed using an adjusted version of the short (18-item) version of the Self-Management Ability Scale (SMAS-S) [61]. The SMAS-S assesses the following six self-management abilities: (1) the ability to take initiative, (2) the ability to invest in those resources that create long-term benefits, (3) the ability to ensuring multifunctionality of resources (gaining and maintaining resources or activities that serve multiple gains), (4) the ability to maintain a variety in resources (gaining and maintaining various resources), (5) the ability to maintain a positive frame of mind, and (6) self-efficacy (the ability to gain and maintain a belief in personal competence to achieve well-being). While the original instrument contains six answering categories we reduced the number of response categories for five subscales from six to four to make completion of the instrument less complex. A higher score implies better self-management abilities. The item ‘when things go against you, how often do you think that it could always be worse?’ proved to be problematic during validation which is why we removed this item from the Turkish version of the instrument. The SMAS-S proved to be a reliable instrument as the Cronbach’s alpha based on the six subscales was 0.92.

Concerning background characteristics, respondents were asked to report the highest educational level completed in the Netherlands or abroad, with the option to select ‘no schooling’ or to write in another response for unlisted forms of schooling. This variable was dichotomised as low (completion of elementary school or less) and high (more than elementary school). Respondents were also asked about their marital status (married, divorced, widowed, single, or cohabitating). This variable was dichotomised as divorced, single, or widowed and married. Finally, we asked respondents to report their age, gender, and number of chronic conditions experienced in the past 12 months.

### Analyses

The characteristics of the study sample were examined using descriptive statistics. Differences in acculturation and self-management abilities between sub-groups (men and women, those with high and low educational levels, those living alone and those who were married, and those with and without multimorbidity) were tested using independent-samples *t* tests and chi-squared tests. Bivariate associations of variables expressing background characteristics, acculturation, and self-management abilities were examined. Regression analyses were then performed to identify relationships among background characteristics, acculturation, and self-management abilities among older Turkish immigrants in Rotterdam, the Netherlands.

## Results

About half (47.6%) of the respondents were women. The average age of respondents was 72.90 [standard deviation (SD), 5.02; range, 66–95] years (Table 1). Most (80.3%) respondents had low educational levels, and 69.4% had multimorbidity. About one-third (34.1%) of respondents used the integration acculturation strategy, 24.7% used the separation strategy, 23.0% used the marginalisation strategy, and 18.2% used the assimilation strategy.

**Table 1:** Descriptive statistics for older Turkish immigrants (*n* = 680)

Characteristic	Range	% or mean (SD)
Sex (female)		47.6%
Age (years)	66–95	72.90 (5.02)
Marital status (single/widowed)		28.7%
Education (low)		80.3%
Number of chronic diseases	0–10	2.68 (1.87)
Co−/multi-morbidity		69.4%
Self-management abilities	1–4	2.52 (0.62)
Attachment to the Turkish culture	1–5	3.92 (0.69)
Attachment to the Dutch culture	1–5	3.19 (0.77)
Integration acculturation strategy		34.1%
Assimilation acculturation strategy		18.2%
Separation acculturation strategy		24.7%
Marginalisation acculturation strategy		23.0%

*SD* standard deviation

Tables 2 and 3 show differences in self-management, acculturation strategies, and attachment to the Dutch and Turkish cultures across sub-groups of older Turkish immigrants. Compared with men, women used the separation strategy more often (33.6% vs. 16.7%; *p* < 0.001) and the integration (29.3% vs. 38.5%; *p =* 0.015) and assimilation (13.5% vs. 22.4%; *p* = 0.004) strategies less often. No difference in the use of the marginalisation strategy was found between men and women. Compared with those with higher education levels, less-educated respondents used the separation strategy more often (28.4% vs. 10.7%; *p* < 0.001) and the integration strategy less often (31.0% vs. 45.8%; *p* = 0.002). Compared with married individuals, single respondents used the separation strategy more often (31.7% vs. 22.1%; *p =* 0.015) and the integration strategy less often (25.1% vs. 37.8%; *p =* 0.002). Compared with respondents with multimorbidity, those without multimorbidity used the integration strategy more often (40.5% vs. 31.4%, *p* = 0.002; Table 2).

**Table 2:** Differences in acculturation strategies between older Turkish sub-groups (*n* = 680)

	Gender (%)			Educational level (%)			Marital status (%)			Multimorbidity (%)		
Acculturation strategy	Male	Female	*χ* ^2^	High	Low	*χ* ^2^	Single	Married	*χ* ^2^	No	Yes	*χ* ^2^
Integration	38.5%	29.3%	0.015	45.8%	31.0%	0.002	25.1%	37.8%	0.002	40.5%	31.4%	0.027
Assimilation	22.4%	13.5%	0.004	22.9%	17.0%	0.127	16.4%	18.6%	0.569	19.5%	17.6%	0.576
Separation	16.7%	33.6%	< 0.001	10.7%	28.4%	< 0.000	31.7%	22.1%	0.015	21.1%	26.3%	0.192
Marginalisation	22.4%	23.7%	0.708	20.6%	23.7%	0.487	26.8%	21.5%	0.176	18.9%	24.7%	0.123

Results are based on two-sided tests

**Table 3:** Differences in self-management and attachment to the Dutch and Turkish cultures across older Turkish sub-groups (*n* = 680)

	Gender				Educational level				Marital status				Multimorbidity			
	Male	Female	*t*	*p*	High	Low	*t*	*p*	Single	Married	*t*	*p*	Yes	No	*t*	*p*
Self-management	2.57 (0.61)	2.45 (0.64)	−2.383	0.017	2.64 (0.59)	2.48 (0.63)	−2.537	0.011	2.43 (0.65)	2.55 (0.61)	−2.170	0.030	2.42 (0.62)	2.74 (0.59)	−5.972	< 0.001
Attachment to the Dutch culture	3.34 (0.72)	3.03 (0.80)	−5.214	< 0.001	3.49 (0.78)	3.11 (0.75)	−5.048	< 0.001	3.00 (0.86)	3.27 (0.72)	−3.660	< 0.001	3.12 (0.78)	3.37 (0.74)	−3.728	< 0.001
Attachment to the Turkish culture	3.87 (0.70)	3.97 (0.68)	1.832	0.067	3.88 (0.72)	3.94 (0.67)	0.965	0.335	3.90 (0.72)	3.93 (0.68)	−0.466	0.641	3.91 (0.72)	3.95 (0.62)	−0.713	0.476

Values are presented as the mean (standard deviation). Results are based on two-sided tests

Women were poorer self-managers than men [2.45 (SD 0.64) vs. 2.57 (SD 0.61); *p =* 0.017], more-educated respondents were better self-managers than those with less education [2.64 (SD 0.59) vs. 2.48 (SD 0.63); *p =* 0.011], married people had significantly higher SMAS-S scores than did single individuals [2.55 (SD 0.61) vs. 2.43 (SD 0.65); *p =* 0.030], and those with multimorbidity were poorer self-managers than those without multiple chronic diseases [2.42 (SD 0.62) vs. 2.74 (SD 0.59); *p <* 0.001; Table 3]. The same patterns were found for attachment to the Dutch culture, with significantly higher scores among men, more-educated respondents, married individuals, and respondents without multimorbidity.

The results of the bivariate correlation analyses are shown in Table 4. A positive relationship was found between older age and attachment to the Turkish culture (*r* = 0.08, *p* < 0.05). In accordance with the differences found between groups, attachment to the Dutch culture was related negatively to female gender (*r* = − 0.20, *p* < 0.001), single status (*r* = − 0.15, *p* < 0.001), low educational level (*r* = − 0.20, *p* < 0.001), and multimorbidity (*r* = − 0.15, *p* < 0.001). A weak positive relationship was found between attachment to the Turkish culture and attachment to the Dutch culture (*r* = 0.09, *p* < 0.05). Self-management was related negatively to female gender (*r* = − 0.10, *p* < 0.05), older age (*r* = − 0.10, *p* < 0.01), single status (*r* = − 0.09, *p* < 0.05), low educational level (*r* = − 0.10, *p* < 0.01), and multimorbidity (*r* = − 0.32, *p* < 0.001). Positive relationships were found between self-management and attachment to the Turkish (*r* = 0.16, *p* < 0.001) and Dutch (*r* = 0.26, *p* < 0.001) cultures. The Fisher test revealed that the correlation coefficient for attachment to the Dutch culture was significantly higher than that for attachment to the Turkish culture (*z*-score = 1.93, *p* = 0.027).

**Table 4:** Associations between background characteristics, attachment to the Dutch and Turkish cultures, and self-management abilities (*n* = 680)

Characteristic	1	2	3	4	5	6	7
1. Sex (female)							
2. Age (years)	−0.05						
3. Marital status (single/widowed)	0.41^***^	0.14^***^					
4. Education (low)	0.25^***^	0.05	0.12^**^				
5. Multimorbidity	0.21^***^	0.06	0.12^**^	0.21^***^			
6. Attachment to the Turkish culture	0.07	0.08^*^	−0.02	0.04	−0.03		
7. Attachment to the Dutch culture	−0.20^***^	− 0.07	−0.15^***^	− 0.20^***^	−0.15^***^	0.09^*^	
8. Self-management abilities	−0.10^*^	−0.10^**^	− 0.09^*^	−0.10^**^	− 0.32^***^	0.16^***^	0.26^***^

***p < 0.001, **p < 0.01, *p < 0.05

The results of the multivariate regression analyses are shown in Table 5. Self-management was associated negatively with older age (β = − 0.09, *p* = 0.016) and multimorbidity (β = − 0.18, *p <* 0.001), and positively with attachment to the Turkish (β = 0.15, *p* < 0.001) and Dutch (β = 0.20, *p* < 0.001) cultures.

**Table 5:** Results of multivariate regression analyses investigating relationships between background characteristics, attachment to the Dutch and Turkish cultures, and self-management of older Turkish immigrants in Rotterdam, the Netherlands

Characteristic	Self-management	
	β	*p*
Sex (female)	−0.02	0.635
Age (years)	−0.09	0.016
Marital status (single/widowed)	−0.01	0.871
Education (low)	−0.02	0.606
Multimorbidity	−0.18	< 0.001
Attachment to the Dutch culture	0.20	< 0.001
Attachment to the Turkish culture	0.14	< 0.001

## Discussion

The results of this study demonstrate the importance of attachment to both the Turkish and Dutch cultures for the self-management abilities of older Turkish immigrants in the Netherlands. Slightly stronger relationships were found between self-management and attachment to the Dutch culture than attachment to the Turkish culture. Cultural values may be conveyed through older people’s self-management abilities. In the Netherlands (as in other Western countries), engagement in self-management activities and the strengthening of self-management abilities have long been employed as effective preventive tools among chronically ill (and frail) older people [62]. Dutch society has changed from a demographically young welfare state to an ageing society with increasing individual responsibility [63]. Research has shown that Arabic- and Turkish-speaking communities initiate participation in self-management activities less often than do native speakers [64]. Muslim people are known to respond to illness and death with patience, meditation, and prayer, and may therefore turn more often to spiritual or religious coping strategies instead of working on self-management abilities [65]. Therefore, we hypothesise that Turkish migrants who follow the integration and assimilation acculturation strategies (both characterised by attachment to the Dutch culture) are better self-managers, not only because the skills needed to integrate contribute to self-management, but also because they fit better in the Dutch culture, in which self-management is embedded more strongly.

Less attachment to Dutch culture was observed among women, single respondents, less-educated individuals, and those with multimorbidity. Men, more-educated respondents, married individuals, and those without multimorbidity used the integration acculturation strategy more often, whereas women, less-educated respondents, and single individuals used the separation strategy more often. Women, single individuals, less-educated respondents, and those with multimorbidity reported poorer self-management abilities. Research among older and chronically ill Dutch people (e.g. [14, 15, 66] has shown that single and less-educated individuals are worse self-managers than are those who are married and more educated. In contrast to the finding that older Turkish women are worse self-managers than their male counterparts, older Dutch women are generally better self-managers than older Dutch men [13, 66]. Older Turkish women may have fewer opportunities to engage in self-management behaviours and have to rely more on relatives and the government. For example, Staring et al. [67] showed that Turkish women living in the Netherlands experience strict social control, which limits their ability to seek out contacts outside the Turkish community. The importance of attachment to the Dutch culture (using the integration or assimilation acculturation strategy) for people’s self-management abilities observed in this study may explain why Turkish women were poorer self-managers than were Turkish men. In multivariate analyses that took attachment to the Dutch and Turkish cultures into account, older age and multimorbidity were the only background characteristics that remained associated (negatively) with Turkish immigrants’ self-management abilities. Van Houtum et al. [68] clearly showed that deteriorating health resulting from chronic illness (es) led to increased self-management support needs over time. Health care for people with (multiple) chronic diseases often focuses only on physical health and clinical outcomes (e.g. 14, 15); older Turkish immigrants with multimorbidity may benefit more from health care supporting their broader self-management abilities.

The limitations of this study should be taken into account when interpreting our findings. First, although the response rate of 32% seems low, it is similar to those achieved in other surveys of the same population [69]. We first invited respondents to fill in the questionnaire via mail, and then made a minimum of two personal contact attempts at respondents’ homes. Previous research, however, has indicated that a minimum of six contact attempts is necessary to achieve an optimal response [5]; this approach was not feasible for the current study. Second, because of the study’s cross-sectional design, we were unable to draw causal conclusions. Longitudinal data are needed to investigate the relationships between acculturation and self-management abilities over time. For example, although we found that multimorbidity was related negatively to the self-management abilities of older Turkish immigrants, this relationship is expected to be dynamic, as poor self-managers probably have a greater chance of becoming chronically ill. Third, the current study was limited to elderly Turkish residents of Rotterdam. A similar study conducted among Moroccans, the second largest migrant group in Rotterdam, would be of interest. Given that acculturation is not the responsibility of migrants solely but rather a result of a mutual dynamic interaction between migrants and the host country a better understanding of the acculturation strategies among various migrants within a country will help identify areas for improvement. In addition, as acculturation strategies and self-management abilities are expected to vary across cultures and countries, comparative studies should be conducted among (im) migrant elders in other countries. Furthermore, we subjected the home and host culture scales to a median split to obtain the four acculturation strategies, following previous research [36, 58–60]. We did not use norm scores to determine the actual acculturation strategy used by each individual. We are therefore not sure whether the acculturation strategy data truly reflect relevant differences in these strategies between groups. Further research is needed to develop norm scores. Fourth, we did not include number of years in the host country. We do know that Turkish older people in the Netherlands are mainly first generation migrants (they were born in Turkey). Most of the men moved to the Netherlands just before 1973 and their families often joined them a couple of years later. This means that most of the Turkish older people live in the Netherlands for over 40 years [70]. Finally, we included four acculturation strategies only. Other theories such as social adaption [71, 72] (the adjustment of individual and group behaviour to conform with the prevailing system of norms and values in a given society, class, or social group) may be interesting to include in future research as well.

From the results of this study, we conclude that especially attachment to the Dutch culture matters for the self-management abilities of older Turkish immigrants in the Netherlands. Given the high prevalence of chronic diseases in this population, investment in their self-management abilities is expected to be beneficial. Special attention is needed for women, single individuals, less-educated people, and those with multimorbidity. Interventions aiming to better integrate these groups into Dutch society are also expected to be beneficial for their self-management abilities.

## Data Availability

The datasets analysed during the current study are available from the corresponding author on reasonable request.

## Abbreviations

CCMO: Central Committee on Research Involving Human Subjects
D-PAS: Dutch Psychological Acculturation Subscale
SMAS-S: Self-Management Ability Scale
T-PAS: Turkish Psychological Acculturation Subscale
